# Proton Pumps: Molecular Mechanisms, Inhibitors and Activators of Proton Pumping

**DOI:** 10.3390/ijms24109070

**Published:** 2023-05-22

**Authors:** Sergey A. Siletsky

**Affiliations:** Belozersky Institute of Physical-Chemical Biology, Lomonosov Moscow State University, 119991 Moscow, Russia; siletsky@belozersky.msu.ru

Protein molecular machines, also known as proton pumps, are the most important element of biological membranes. These are membrane proteins that are widely represented and distributed in all groups of living organisms (including some viruses). They have the ability to create and maintain an electrochemical proton gradient by transferring protons from one side of the membrane to the other. Proton pumps are divided into various large classes, which differ in their use of different energy sources, each having different polypeptide composition and evolutionary origin.

The source of free energy for pumping protons in protein pumps can be: the chemical energy of energy-rich metabolites (f.e., in proton ATPases), electron transfer energy from compounds with lower redox potential (f.e., in mitochondrial respiratory chain complexes), and light energy (f.e., in retinal proteins). The transfer of a proton in proton pumps is usually electrogenic. However, there are also no less significant, and perhaps even more significant non-electrogenic proton pumps, such as hydrogen–potassium ATPase or H^+^/K^+^ ATPase of the gastric mucosa, which is primarily responsible for the acidification of stomach contents.

The new Special Issue entitled “***Proton pumps: molecular mechanisms, inhibitors and activators of proton pumping***” includes a total of six contributions: four original articles and 2 reviews. These articles and reviews provide new information relating to proton pumps, starting with an understanding of the basics of the mechanism of reactions catalyzed by them, their significance in cellular physiology and the molecular mechanisms of intracellular signaling, and ending with their applied use in medicine. Despite the modest number of contributions, they touch on a wide range of both fundamental and applied issues and provide new information: on the molecular mechanisms and catalytic features of specific protein proton pumps (in particular, cytochrome oxidase and ATP synthetase); on the features of cellular physiology and the regulation and mechanisms of signal transduction involving proton pumps; and on molecular medical research of the use of medicines—inhibitors of the proton pump of the stomach H^+^/K^+^ ATPase.

The review of Siletsky S.A. and Borisov V.B. [1] analyzes recent structural and functional studies of oxygen reduction intermediates in the active sites of terminal respiratory oxidases, the features of catalytic cycles and the properties of the active sites of these enzymes. The terminal respiratory oxidases include two main groups of functionally similar, but structurally and evolutionarily strikingly different superfamilies: heme-copper oxidases (HCOs, including cytochrome oxidase (COX) of mitochondria) and *bd*-type cytochromes. All of them are united by a catalytic reaction of the four-electron reduction of oxygen into water, which proceeds without the formation and release of potentially dangerous reactive oxygen species (ROS) from active sites. These membrane enzymes of eukaryotes and prokaryotes transform the energy of chemical bonds released during the transfer of electrons from cytochromes or quinols to molecular oxygen into a transmembrane proton gradient. In contrast to *bd*-type oxidases, HCOs generate the proton motive force not only by the transfer of electrons and protons to the catalytic center from different sides of the membrane but also due to the unique ability for redox-coupled directed proton pumping through the membrane. To date, three-dimensional structures with atomic resolution of members of all major groups of terminal respiratory oxidases, heme-copper oxidases and *bd*-type cytochromes have been obtained. The structures of representatives of heme-copper oxidases of the C family and cytochromes bd (in the latter case, literally in the last few years) were determined the most recently. At the same time, both of these groups of the terminal oxidases have enormous biomedical significance, since they allow microorganisms to adapt to low-oxygen conditions, survive in chemically aggressive environments and acquire antibiotic resistance.

However, the presence of the structure gives a static picture, whereas the catalytic cycle consists of many small changes in the active centers and the structure of the intra-protein proton-conducting pathways, which remain largely unexplored in detail. The mechanism of catalysis and energy conversion during proton pumping in COX is one of the most significant challenges in molecular bioenergetics and membranology. It is impossible to fully describe it without clarifying the detailed structure of the individual intermediate states of the catalytic cycle and the transitions between them, as well as the individual stages of electron and proton transfer with adequate time resolution. Despite the progress made in recent years in this direction, there are myriad unresolved issues that are waiting to be solved through new research.

The research article by Sztachova T. et al. (corresponding authors Jancura D. and Fabian M. [2]) is devoted to elucidating the structure of the intermediate states within the catalytic cycle of cytochrome oxidase from bovine mitochondria. One of the most important approaches in the study of intermediate states of the catalytic cycle of cytochrome oxidase is the study of complexes of the oxygen-reducing binuclear center (the so called BNC, consisting of the heme *a*_3_ iron and copper atom of Cu_B_) with molecules, simulating the states of intermediate oxygen reduction. Of these molecules, hydrogen peroxide occupies a special place. Depending on the concentration and pH, hydrogen peroxide transforms BNC into a number of different intermediates of the catalytic cycle (the so-called P, F states and a new recently discovered at acidic pH intermediate state F*.

This work is devoted to a comprehensive study of this intermediate state (F*) using several methodological approaches. In the catalytic cycle of cytochrome oxidase, the P state precedes the F state and is characterized by the presence of a tyrosine radical near BNC. During the formation of the P state, an almost instantaneous four-electron reduction of oxygen in BNC occurs with the formation of four-electron vacancies, due to which the release of intermediate forms of oxygen reduction (reactive oxygen species, ROS) is excluded. The fourth electron comes from tyrosine, which becomes a radical. In this study, the formation of the radical in the F* state of the purified bovine COX was detected, and its lifetime at the catalytic center was examined. The data obtained by the application of several experimental approaches (including isothermal titration calorimetry, electron paramagnetic resonance, and electronic absorption spectroscopy) convincingly prove the production of the radical at the catalytic center in the F* form. The nature, physicochemical characteristics and differences from other intermediate states of this radical in the catalytic cycle of cytochrome oxidase are discussed.

The research article by Iván Pérez et al. (corresponding author Michael Börsch [3]) is devoted to elucidating the mechanism of proton-pumping FoF1-ATP synthase using a complex of the most modern methods of molecular biophysics and chemical kinetics, allowing researchers to trace the catalytic transformations in one single enzyme molecule. FoF1-ATP synthases provide the energy of ATP molecules to cellular needs, being membrane enzymes and using the energy of proton transfer along the membrane potential gradient in mitochondria, chloroplasts and most bacteria for the synthesis of ATP from ADP and phosphate. Traditional kinetic approaches in enzymology and biophysics operate with an ensemble of molecules. Therefore, they allow us to obtain only an average picture of the mechanism of the enzymes. Especially important is the lack of information about the individual behavior of enzyme molecules when studying the mechanisms of inhibition, since in this case, it is often impossible to distinguish the inhibition of the entire ensemble of molecules, to the same extent, from the shutdown of part of the ensemble, while maintaining the full activity of the other part of the ensemble of molecules. The specific objective of this study was to understand the mechanism of ADP-inhibited ATP hydrolysis in single proton-pumping FoF1-ATP synthase trapped in solution. ATP synthase uses a mechanism rotating inside the membrane to interface proton flow through the membrane down the gradient of the membrane potential in the Fo part of the enzyme with catalytic transformations of ADP and phosphate to ATP in the extra-membrane part (F1) of the enzyme. The authors investigated the effect of changes in ADP concentration on the rate of ATP hydrolysis by single FoF1-ATP synthases in proteoliposomes. They were able to track the catalytic cycles of single enzyme molecule following each other by registering the rotation of fluorescently labeled intramembrane subunits of the rotary part of the enzyme using single-molecule Förster resonance energy transfer. Thanks to the unique combination of the most modern methods used, the authors were able to establish the details and kinetics of the mechanism of inhibition of this enzyme at the level of a single molecule.

The review of Chen J et al. (corresponding author Gao H. [4]) discusses an extremely important problem of cellular physiology, in which heme-copper oxidases are involved. These are the effects of low molecular regulators, such as nitrogen oxides, the sensitivity to which is inherent in many heme-containing enzymes, including heme-copper oxidases. Among them, nitrite and nitric oxide are of the greatest interest, and this review is devoted to their complex interplay with heme-copper oxidases. Much attention is paid to the attempt to separate (which is one of the many obvious advantages of the review) and characterize the role of heme-copper oxidases in the transformation of nitrogen oxides, on the one hand, and the inhibitory effects of nitrogen compounds on heme-copper oxidases on the other. The authors of the review rightly and argumentatively point out that the importance of nitrite in the physiology of living organisms remains undervalued and unrecognized, since the idea that nitrite influences through the formation of NO is in some way a dogma. However, new data have been presented in recent years showing that nitrite and NO can be perceived by bacterial cells as two different nitric oxides. If NO interacts with most redox-sensitive proteins, then nitrite exhibits high specificity. In particular, NO presumably interacts with all hemoproteins (including various cytochromes), whereas nitrite specifically inhibits heme-copper oxidases. The authors pay great attention to the variety of strategies for reacting and combating bacteria with nitrites and NO in bacteria based on sensory proteins. They indicate possible unexplored features of these sensory hemoproteins that allow them to distinguish nitrogen compounds, with the possibility of using this knowledge to advance nitrites along the path from being perceived by everyone as a toxin to being used in therapy, in addition to NO, which has been used in clinical settings for decades.

A very interesting work, the research article of Malgorzata Janicka et al. is devoted to the study of important problems of cellular physiology, namely, deciphering the ways of transmitting regulatory signals that affect the activity of proton ATPase in normal and stress conditions, including those caused by heavy metals [5]. An important direction in the study of proton pumps is their connection and the effects of heavy metal ions on them. Heavy metal ions have different effects on proton pumps. In addition to the inhibitory effect of zinc ions, cadmium, etc., directly on the proton-conducting pathways when binding on the surface of the protein, a more complex and multifaceted picture is revealed when considering these effects at the cellular level. It transpires that these effects at the cellular level can be much more diverse and multidirectional. Around 90% of all arable lands are subjected to environmental stresses such as drought, low or high temperature, salinity, heavy metal exposure and others. Cadmium ions are easily taken up by plant roots and can accumulate for long periods of time inside a food chain (in plants and animals). Unlike a number of other heavy metals, cadmium is not a catalyst in the Fenton reaction and does not directly cause the formation of hydrogen peroxide. However, its presence is indirectly accompanied by oxidative stress and the formation of peroxide and NO. Flavin adenine dinucleotide (FAD-dependent polyamine oxidases, PAO) and copper amine oxidases (CuAO, also called diamine oxidases, DAO) are responsible for the oxidation of polyamines in plants, which occurs with production of H_2_O_2_. A new signaling pathway is proposed by the authors, some of the elements of which are the activation of the H^+^-ATPase of the plasma membrane (the most exposed plant membrane to the external environment) through DAO, which forms peroxide and NO. A number of proofs of the existence of the new signaling pathway using a specific DAO inhibitor have been carried out.

Of particular importance is the application of the knowledge gained relating to molecular proton pumps in molecular medical science and in practical medicine. This area is a combination of fundamental and applied research. An example of this is the research article by Russo M. et al. (corresponding author Tursi A. [6]) that is devoted to the medical aspects of the use of proton pump inhibitors in clinical practice. Proton pump inhibitors (PPIs) are first-choice drugs for the treatment of acid-related disorders, such as gastroesophageal reflux disease and peptic ulcer disease, due to selective inhibition of the proton pump in the stomach (H^+^/K^+^ ATPase). Inappropriate prescription of PPIs has been widely reported, often lacking initial exclusion of *Helicobacter pylori* (HP) infection and evaluation of gastric functional status. The aim of this study was to evaluate the utility of gastric functional tests to define the acid output, as well as HP status, in order to better direct PPI therapy prescription. The gastric function test constitutes a non-invasive determination of four serum parameters. These four biomarkers include pepsinogen I (PGI), pepsinogen II (PGII), amidated gastrin-17 (G17) and HP antibodies. The interpretation of four serum parameters is based on the combination of values included in the panel. A total number of 2583 patients undergoing gastric function test were evaluated. This study revealed that the use of a panel composed of four serological markers (PGI, PGII, G17, anti-HP IgG) is a valid tool to be used as a first-level test to be carried out before undertaking a course of therapy with PPIs, to evaluate the state of the mucosa, HP infection and, more generally, the appropriateness of prescribing these drugs. As a result of the conducted research, practical recommendations for clinical doctors can be developed, and the strategy may be adopted within the diagnostic algorithms and in prescribing guidelines, thus avoiding unnecessary and harmful side effects relating to PPI therapy.
